# Changes in Tumor-Infiltrating Lymphocytes and Vascular Normalization in Breast Cancer Patients After Neoadjuvant Chemotherapy and Their Correlations With DFS

**DOI:** 10.3389/fonc.2019.01545

**Published:** 2020-01-22

**Authors:** Qiong Wang, Qun Xiang, Lan Yu, Ting Hu, Yangyang Chen, Jue Wang, Xiu Nie, Jing Cheng

**Affiliations:** ^1^Cancer Center, Union Hospital, Tongji Medical College, Huazhong University of Science and Technology, Wuhan, China; ^2^Department of Pathology, Union Hospital, Tongji Medical College, Huazhong University of Science and Technology, Wuhan, China

**Keywords:** breast cancer, tumor-infiltrating lymphocyte, neoadjuvant chemotherapy, microvessel pericyte coverage index, FOXP3+ Tregs

## Abstract

**Objective:** Changes in the number of various tumor-infiltrating lymphocytes (TILs) and degrees of vascular normalization in breast cancer (BC) patients after neoadjuvant chemotherapy (NAC) were analyzed to screen key factors that can predict the prognosis.

**Methods:** HE-stained sections were used to assess the degree of TILs infiltration; immunohistochemically stained sections were used to assess the infiltration of CD8+, CD4+, FOXP3+ Tregs and the expression of PD-L1; immunofluorescence-stained sections were used to assess the microvessel density (MVD) and microvessel pericyte coverage index (MPI). The expression of them before NAC were compared with those after NAC, and correlations between changes in these parameters and the pathological complete remission (pCR) and DFS of BC patients were analyzed.

**Results:** After NAC, the percentage of patients with enhanced sTILs in the pCR group was significantly higher than that in the Non-pCR group (*P* < 0.05). Univariate and multivariate analyses showed that the number of FOXP3+ Tregs and MPI before NAC were correlated with pCR (*P* < 0.05). Survival analysis showed that the DFS of BC patients with reduced FOXP3+ Tregs was significantly better than that of patients with elevated FOXP3+ Tregs (*P* = 0.029). The sTILs count and MPI were significantly higher in primary tumors than lymph nodes (*P* < 0.05).

**Conclusion:** After NAC, the reduced infiltration of FOXP3+ Tregs was correlated with an improvement in DFS in BC patients. Changes in the number of FOXP3+ Tregs and the MPI may be used as prognostic markers for BC patients.

## Introduction

Among females, breast cancer (BC) is the malignant tumor with the highest incidence (1) and seriously threatens the health of women worldwide (2). The incidence of BC in China increases yearly, and the mortality rate remains high (3). The primary reason for failure of BC treatment is relapse and metastasis of BC after treatment (4, 5). Neoadjuvant chemotherapy (NAC) is currently the main treatment for locally advanced BC and it can be used to detect the sensitivity of tumor cells to anticancer treatment and to guide the follow-up treatment (6). Our understanding of these patients is still insufficient; recurrence and metastasis still occur in patients who achieve pCR after NAC.

Tumor-infiltrating lymphocytes (TILs) are the most important immune cells in the tumor microenvironment (TME) (7). For BC, according to the distribution of TILs in tumor tissues, TILs can be divided into intratumoral TILs (iTILs), and stromal TILs (sTILs). The International TILs Working Group recommends the use of sTILs to assess TILs in BC (8). The traditional view is that BC lacks immunological activity (9). However, a growing body of evidence suggests that pretreatment of sTILs in BC patients can predict treatment response and is correlated with prognosis (10, 11). Therefore, sTILs may become a biomarker for efficacy prediction and the prognosis of BC patients (12, 13). Denkert divided BC patients into three groups according to sTILs infiltration in tumor tissues: low sTILs infiltration (0–10%), medium sTILs infiltration (11–59%) and high sTILs infiltration (60–100%), their studies suggested that high sTILs infiltration can predict the NAC response of all BC subtypes and is correlated with the survival benefit of Her2-positive BC and triple-negative breast cancer (TNBC) (14). In addition to TILs, programmed death ligand 1 (PD-L1) in the TME is widely studied (15). The expression of PD-L1 is closely correlated with the presence of TILs. Programmed death-1 (PD-1) expressed by TILs binds to PD-L1 expressed on tumor cells to allow tumors to escape anti-tumor immunity in the TME of BC (16).

Another key factor in the TME of BC is vascular normalization, which involves increased pericyte coverage, improved tumor vessel perfusion, reduced vascular permeability, and consequently mitigated hypoxia (17). Vascular normalization can promote T lymphocyte infiltration, and interferon-γ-secreting T helper 1 (Th1) cells are the major cell population associated with vascular normalization, which increases pericyte coverage and tumor vessel normalization (18, 19). Microvessel density (MVD) is an important indicator for evaluating tumor angiogenesis (20). The microvessel pericyte coverage index (MPI) is an important indicator reflecting vascular normalization. MPI means the ratio of microvascular density covered by pericytes to total microvascular density in tumor tissues. The MPI of most tumor blood vessels is low, and the function of the tumor vascular bed is not mature (21). The currently available studies on tumor vascular normalization and TILs are primarily fundamental studies that have not been conducted in clinical practice.

In the present study, changes in sTILs, PD-L1 expression and vascular normalization were examined in BC patients before and after NAC, the effect of NAC on the TME of BC was investigated, and its correlation with the post-NAC pCR rate and disease-free survival (DFS) was analyzed to find appropriate predictive and prognostic biomarkers for treatment of BC.

## Materials and Methods

### Research Subjects

This study included 75 BC patients who were admitted to the Cancer Center, Union Hospital, Tongji Medical College, Huazhong University of Science and Technology, and treated with NAC between November 2013 and December 2018. All patients underwent core needle biopsy at initial diagnosis and were pathologically confirmed as invasive BC. All patients received four or more cycles of NAC, which was a combined chemotherapy using anthracyclines, cyclophosphamide, or taxanes. For patients with HER-2 positive BC, Trastuzumab was used. No other drugs were used as NAC treatment in our study including Pertuzumab and endocrine therapy etc. According to the pathological biopsy report, patients were divided into four molecular subtypes, HER2-positive BC, luminal/HER2-negative BC, luminal/HER2-positive BC, and TNBC. Three weeks after NAC, modified radical mastectomy or breast-conserving surgery for BC was performed in our hospital. After the surgery, the appropriate standard adjuvant therapy (radiotherapy, chemotherapy, targeted therapy, or endocrine therapy) was administered according to patient conditions.

(1) Inclusion criteria:

1. Female patients with preoperative biopsy-confirmed invasive BC;
2. Patients who received 4 or more cycles of preoperative NAC;
3. Patients who underwent modified radical mastectomy or breast-conserving surgery after NAC;
4. Patients who did not have any other immune system diseases and did not use immunosuppressants.

(2) Exclusion criteria:

1. Male BC patients;
2. Patients with second primary cancer at other sites at the time of diagnosis;
3. Patients who received fewer than 4 cycles of preoperative NAC;
4. Patients with incomplete pathological and follow-up data.

### Collection of the Clinical Data

The general clinical data of patients included time of diagnosis, age, diagnosis, pathological data before NAC, NAC regimens, operation time, surgical pathology data, postoperative treatment method, and post-treatment follow-up data. PCR was the primary end point of this study, and pCR was defined as follows: after NAC, no primary invasive cancer component was observed in primary tumors or metastatic lymph nodes, or only components of carcinoma *in situ* were observed in primary tumors and metastatic lymph nodes. DFS was the secondary end point of the study and was defined as the time from the date of surgery to the date of disease progression.

Paraffin-embedded specimens from the core needle biopsy before NAC and paraffin-embedded specimens from surgery were collected from the specimen bank of the Department of Pathology in our hospital. For patients who achieved pCR after NAC, paraffin-embedded specimens from the tumor bed were selected. For patients who did not achieve pCR, if the lymph nodes were negative, paraffin-embedded specimens from residual tumors were selected; if lymph nodes were positive, paraffin-embedded specimens from residual tumors and metastatic lymph nodes were both selected. Each paraffin-embedded specimen was continuously sectioned into six pieces, and the thickness of each section was 4 μm. Hematoxylin-eosin (HE), immunohistochemical and immunofluorescence staining were performed.

### HE Staining, Immunohistochemistry, and Immunofluorescence

Paraffin-embedded sections were dewaxed and hydrated, followed by hematoxylin and eosin staining, dehydration, mounting and microscopic observation, and analysis. The prepared paraffin-embedded sections were used to accept immunohistochemistry and immunofluorescence testing. The antibodies used were showed in the Table 1.

**Table 1:** Concentrations of primary antibodies.

**Primary antibody**	**Source**	**Concentration**
CD8 rabbit anti-human polyclonal antibody (17335-1-AP)	Proteintech, US	1:1,000
CD4 mouse anti-human monoclonal antibody	DAKO, Denmark	Ready-to-use
FOXP3 rabbit polyclonal antibody (22228-1-AP)	Proteintech, US	1:1,600
PD-L1 rabbit anti-human polyclonal antibody (28076-1-AP)	Proteintech, US	1:200
CD105 mouse anti-human monoclonal antibody (BM1725)	Boster, Wuhan, China	1:100
NG2 rabbit anti-human monoclonal antibody (ab183929)	Abcam, UK	1:100

### Pathological Evaluation

1. Assessment of sTILs: (1) According to the recommendations of the International TILs Working Group (8), the sTILs score was defined as the area percentage of mononuclear inflammatory cells in the tumor stroma. The sTILs score was set as a continuous variable, and the positive cutoff value was set at 1%. (2) The entire HE-stained section was first observed under low magnification (×10) to evaluate whether the distribution of sTILs was uniform. (3) If the distribution of sTILs was relatively uniform, three high-magnification (×40) views were randomly selected to calculate the mean sTILs count. (4) If the distribution of sTILs was not uniform, the regions with the highest and lowest sTILs numbers were excluded before three high-magnification views were randomly selected to calculate the mean sTILs count.
2. Evaluation of CD8+ T cells, CD4+ T cells, and FOXP3+ Tregs: (1) First, the entire section was evaluated under low magnification (×10) to observe whether the positive cells were uniformly distributed in tumor stroma; (2) if the distribution of positive cells was relatively uniform, three high- magnification views (×40) were randomly selected to count the number of positive cells, and the mean positive cell count was obtained. (3) If the distribution of positive cells was not uniform, the regions with the highest and lowest numbers of positive cells were excluded before three high-magnification views were randomly selected to count the number of positive cells, and the mean positive cell count (unit: /HPF) was obtained.
3. PD-L1 assessment: (1) PD-L1 staining in tumor cells and lymphocytes was first observed under low magnification (×10); samples with any positive staining of tumor cells, lymphocyte membranes and/or lymphocyte cytoplasm were defined as PD-L1 positive. (2) The percentages of PD-L1-positive cells among all tumor cells and lymphocyte regions were determined in three regions with abundant expression of PD-L1 (i.e., hotspots) under high magnification, and the mean value was obtained.
4. MVD and MPI: (1) First, blue fluorescence was selected to observe cell nuclei and locate the tumor area. Red fluorescence was then selected, and the area with the most abundant red staining (i.e., CD105-positive staining) in the tumor region was selected at low magnification (×10) (hotspot) and photographed at high magnification (×200). (2) Green fluorescence was selected and photographed. (3) Blue fluorescence was selected and photographed. (4) Photographs taken in the first 3 steps were merged. (5) A total of 3 hotspot regions were selected for photographing and image merging using the same method. (6) The MVD of CD105-positive microvessels and the number of microvessels covered with NG2 were determined in each merged image. MPI = Number of microvessels covered with NG2/Total number of microvessels ^*^ 100%. Finally, the mean MPI was calculated.
5. Evaluation of sTILs and PD-L1 in lymph nodes: First, tumor cell-rich lymph nodes were selected under low magnification, and all indicators were then evaluated under high magnification according to the interpretation method for tumor tissues.

All sections were read twice, and the second reading was performed 2–4 weeks after the first reading. When differences of more than 10% were observed between the two readings, the sections were read again, and finally, the mean values were taken. For cases that achieved pCR, the TILs count and PD-L1 expression in the tumor bed was evaluated.

### Statistical Analysis

All count data are expressed as the mean ± standard deviation. Pearson's chi-square test or Fisher's exact test was used to compare categorical variables, and a rank sum test was used to compare numerical variables. Univariate and multivariate analyses were performed using binary logistic regression analysis, and the odds ratio (OR), 95% confidence interval (95% CI) and *P*-value were calculated. The Kaplan-Meier method was used for survival analysis. SPSS 24.0 statistical software (IBM Corp. Armonk, New York) was used for all statistical analyses, and GraphPad Prism 7.0 software was used for plotting. A two-tailed test was performed. *P* < 0.05 was considered to indicate a statistically significant difference.

## Results

### Clinical Data of Patients

In total, 75 BC patients were enrolled in this study (Table 2). Seventy-three of them had invasive ductal carcinoma of the breast, one had inflammatory BC, and one had invasive micropapillary carcinoma of the right breast during pregnancy. The median age of all patients was 48 years (29–67 years). Among the molecular subtypes, patients with HER2-positive BC accounted for 28.0% (*n* = 21), patients with luminal/HER2-negative BC accounted for 29.3% (*n* = 22), patients with luminal/HER2-positive BC accounted for 18.7% (*n* = 14), and patients with TNBC accounted for 24.0% (*n* = 18). Before NAC, the clinical stage of BC ranged from II-IV, including 47 cases (62.7%) of stage II patients, 27 cases (36.0%) of stage III patients, and 1 (1.3%) case of a stage IV patient. For the histological grades of BC, patients with Grades II and III accounted for 45.3% and 45.3%, respectively, and 76% of the patients had high levels of Ki-67 expression (Ki-67 ≥ 14%).

**Table 2:** Patients clinical characteristics.

**Parameter**		***N* (%)**
**Age**		
	<50 years	47 (62.7)
	≥50 years	28 (37.3)
**Site**		
	Left	44 (41.3)
	Right	31 (58.7)
**Molecular subtype**		
	Her2 positive	21 (28.0)
	Luminal-Her2 (–)	22 (29.3)
	Luminal-Her2 positive	14 (18.7)
	TNBC	18 (24.0)
**Clinical stage**		
	II	47 (62.7)
	III	28 (37.3)
**T stage**		
	T1	7 (9.3)
	T2	52 (69.3)
	T3	8 (10.7)
	T4	8 (10.7)
**N stage**		
	N0	16 (21.3)
	N1	39 (52.0)
	N2	15 (20.0)
	N3	5 (6.7)
**Tissue stage**		
	I	7 (9.4)
	II	34 (45.3)
	III	34 (45.3)
**Ki67**		
	<14%	18 (24.0)
	≥14%	57 (76.0)
**pCR state**		
	pCR	20 (26.7)
	Non-pCR	55 (73.3)

Among the 75 BC patients, 20 (26.7%) achieved pCR after surgery: 23.8% (5/21) of patients with HER2-positive BC achieved pCR, 38.9% (7/18) of TNBC patients achieved pCR, and 22.2% (8/36) of patients with luminal BC achieved pCR.

### sTIL, CD8+ T Cell, CD4+ T Cell, and FOXP3+ Tregs Counts, PD-L1 Expression, MVD, and MPI in BC Patients Before and After NAC

**Figure 1:**
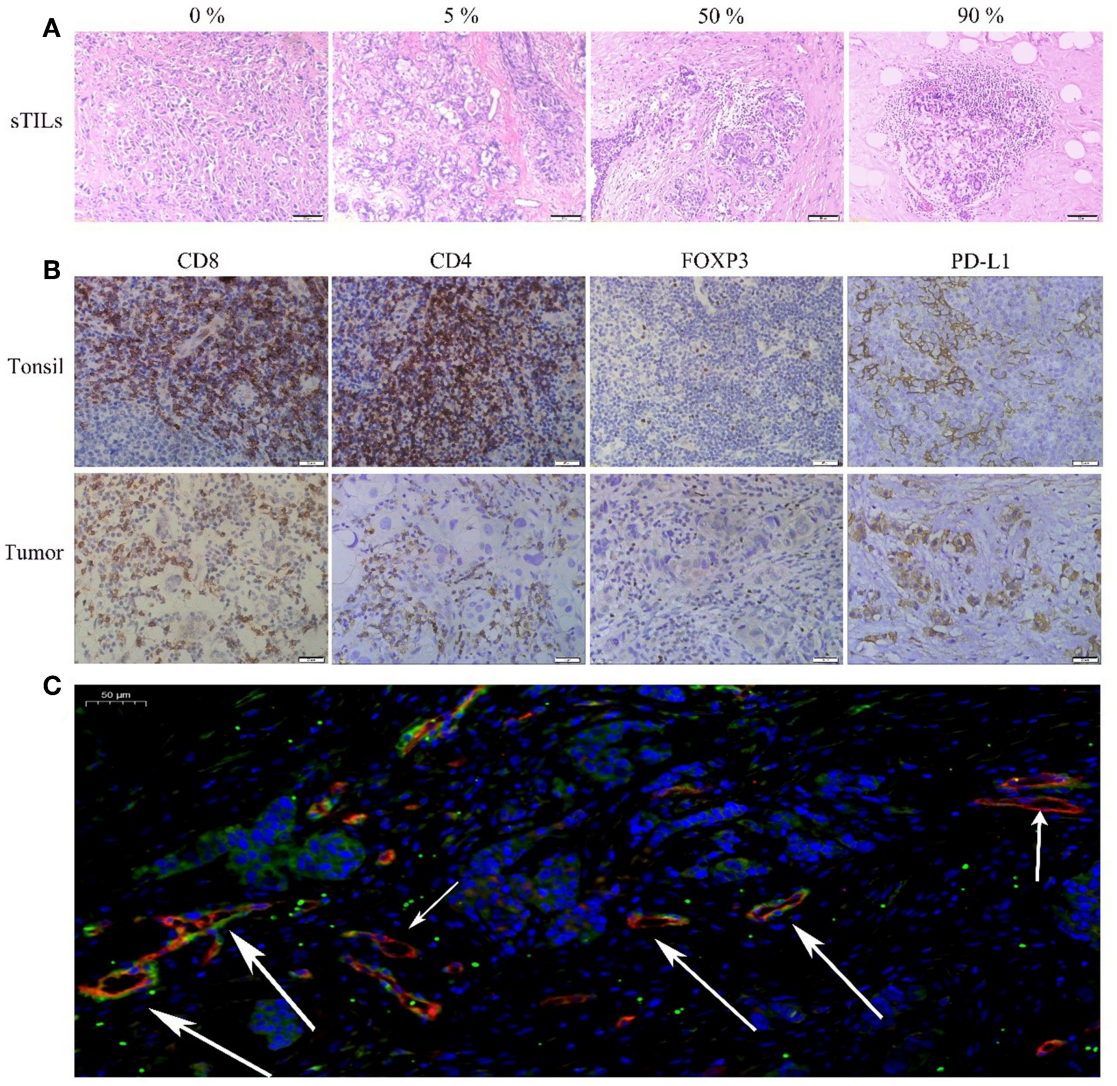
**(A)** HE(x200). **(B)** Immunohistochemical (IHC, x400) CD8+, CD4+, FOXP3+Tregs, and PD-L1 of Tonsil (positive tissue) and breast tumor. **(C)** Immunofluorescence (IF, x20) Blue: DAPI; Red: CD105; Green: NG2 (The short arrow shows microvessels not covered by pericyte cells, the long arrow shows microvessels covered by pericyte cells).

**Figure 2:**
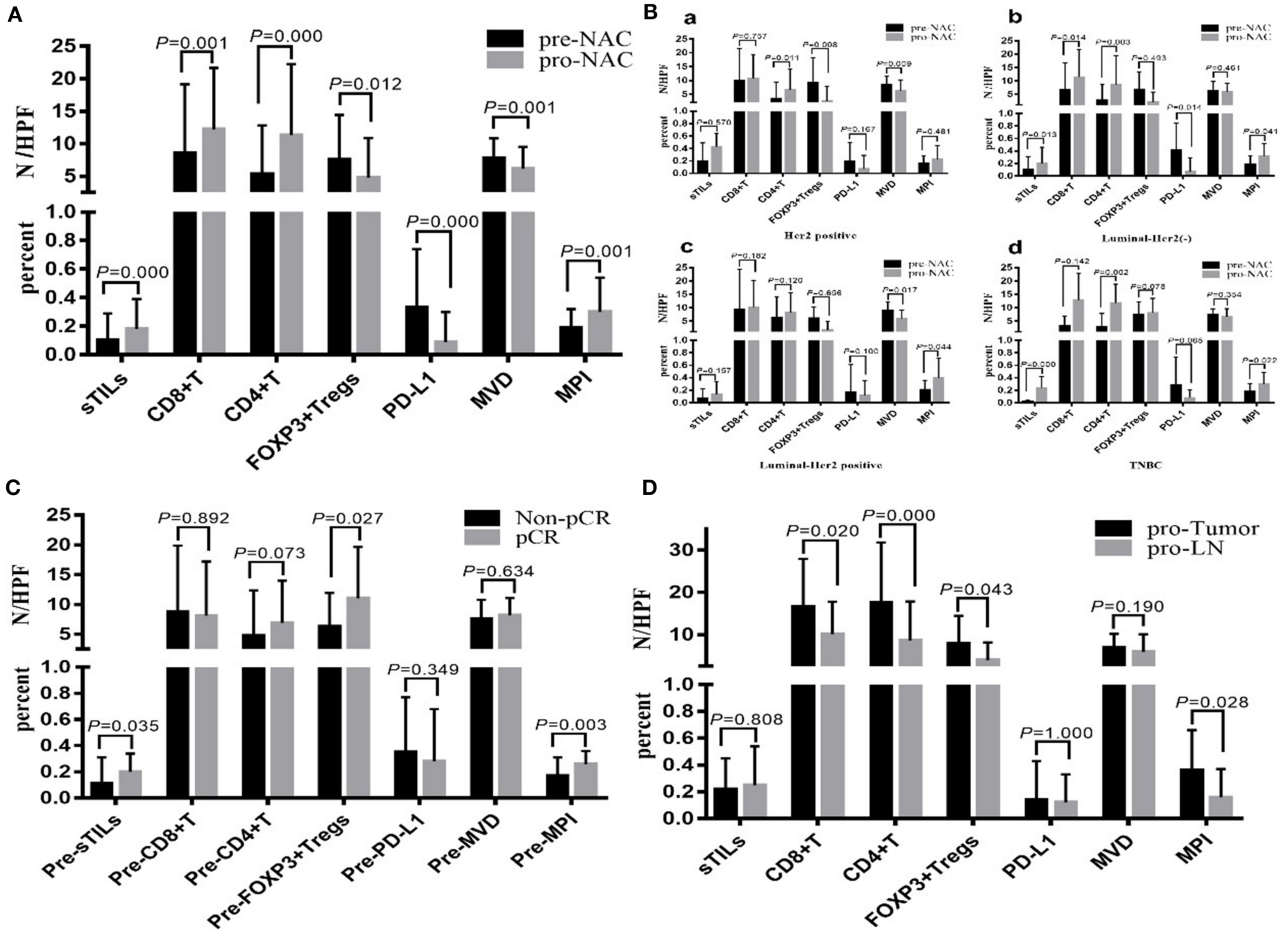
**(A)** Changes of total sTILs, PD-L1, MVD and MPI before and after NAC. **(B)** Changes of sTILs, PD-L1, MVD, and MPI before and after NAC in patients with different breast cancer subtypes: a. Her2 positive; b. Luminal—Her2 (–); c. Luminal-Her2 positive; d. TNBC. **(C)** Comparison of sTILs, PD-L1, MVD, and MPI between Non-pCR group and pCR group before NAC. **(D)** Comparison of sTILs, PD-L1, MVD, and MPI in primary tumor (pro-Tumor) and metastatic lymph nodes (pro-LN) after NAC (*N* = 21).

Figure 1A shows the different degrees of sTILs infiltration in HE-stained sections of tumor tissues. Figure 1B shows the results of immunohistochemistry staining. Figure 1C shows the results of immunofluorescence staining. Based on the number of sTILs, CD8+ T cells, CD4+ T cells, and FOXP3+ Tregs and the PD-L1 expression, MVD, and MPI values before and after NAC, our study found that after NAC, sTILs, CD8+ T cells, CD4+ T cells, and MPI were increased in tumor tissue, and FOXP3+ Tregs, PD-L1 expression, and MVD were decreased in tumor tissue (Figure 2A). Comparison of patients with different molecular subtypes of BC revealed that after NAC, the Her2-positive patients showed significantly enhanced infiltration of CD4+ T cells (*P* = 0.011) and significantly reduced FOXP3+ Tregs and MVD (*P*_FOXP3+Tregs_ = 0.008, *P*_MVD_ = 0.009). However, there was no significant difference in the total number of sTILs and CD8+ T cells, MPI, and PD-L1 expression before and after NAC (Figure 2B). After NAC, patients with luminal/HER2-negative BC showed a significant increase in sTILs, CD8+ T cells, CD4+ T cells and MPI (*P* < 0.05); a significant decrease in PD-L1 expression (*P* = 0.041); and a non-significant decrease in MVD and FOXP3+ Tregs (Figure 2B). After NAC, patients with luminal/HER2-positive BC presented a significant increase in MPI (*P* = 0.044), a significant decrease in MVD (*P* = 0.017), and a non-significant change in the number of sTILs, CD8+ T cells, CD4+ T cells, and FOXP3+ Tregs and in PD-L1 expression (Figure 2B). After NAC, patients with TNBC showed a significant increase in sTILs, CD8+ T cells, CD4+ T cells and MPI (*P* < 0.05) (Figure 2B).

Baseline sTILs and FOXP3+ Tregs and MPI in tumor tissues from BC patients in the pCR group were higher than those in the Non-pCR group (*P*_sTILs_ = 0.035, *P*_Tregs_ = 0.027, *P*_MPI_ = 0.005). There was no significant difference in CD8+ T cells, CD4+ T cells, PD-L1 expression, and MVD between the patients in the pCR and Non-pCR groups (Figure 2C).

From comparison of the post-NAC changes in sTILs, CD8+ T cells, CD4+ T cells, FOXP3+ Tregs, PD-L1 expression, MVD, and MPI between BC patients in the pCR and Non-pCR groups, the percentage of patients with enhanced sTILs, CD8+ T cells, and CD4+ T cells infiltration in the pCR group was found to be significantly higher than that in the Non-pCR group (*P* < 0.05), while the percentage of patients with reduced FOXP3+ Tregs in the pCR group was significantly higher than that in the Non-pCR group (*P* = 0.000) (Table 3).

**Table 3:** Changes of sTILs, PD-L1, MVD, and MPI in non-pCR group and pCR group after NAC.

**Parameter**		**Non-pCR group**	**pCR group**	***P***
**sTILs change**				0.020
	Up	34 (61.8)	19 (95.0)	
	Down	14 (25.5)	1 (5.0)	
	Stable	7 (12.7)	0 (0.0)	
**CD8+T change**				0.037
	Up	33 (60.0)	18 (90.0)	
	Down	14 (25.5)	2 (10.0)	
	Stable	8 (14.5)	0 (0.0)	
**CD4+T change**				0.012
	Up	35 (63.6)	19 (95.0)	
	Down	6 (10.9)	1 (5.0)	
	Stable	14 (25.5)	0 (0.0)	
**FOXP3+Tregs change**				0.000
	Up	25 (45.5)	0 (0.0)	
	Down	22 (40.0)	17 (85.0)	
	Stable	8 (14.5)	3 (15.0)	
**PD-L1 change**				0.627
	Up	13 (23.6)	7 (35.0)	
	Down	29 (52.7)	9 (45.0)	
	Stable	13 (23.6)	4 (20.0)	
**MVD change**				0.478
	Up	16 (29.1)	3 (15.0)	
	Down	37 (67.3)	16 (80.0)	
	Stable	2 (3.6)	1 (5.0)	
**MPI change**				0.557
	Up	36 (65.5)	16 (80.0)	
	Down	16 (29.1)	4 (20.0)	
	Stable	3 (5.5)	0 (0.0)	

### sTILs, CD8+ T Cells, CD4+ T Cells, and FOXP3+ Tregs Counts; PD-L1 Expression; MVD; and MPI in Primary Tumors and Metastatic Lymph Nodes After NAC

Among the 55 patients with BC in the non-pCR group, 25 cases were confirmed by postoperative pathology to have lymph node metastasis. For four patients among the 25 cases, only small specimens, which could not be further divided due to the small amount of metastatic cancer components, could be obtained from postoperative metastatic lymph nodes. Therefore, in this study, the postoperative sTILs count, PD-L1 expression, MVD and MPI in primary tumors and metastatic lymph nodes in 21 patients were compared (Figure 2D). The results showed that CD8+ T cells, CD4+ T cells, FOXP3+ Tregs and MPI in primary tumors were all higher than those in metastatic lymph nodes (*P*_CD8+_ = 0.020, *P*_CD4+_ = 0.000, *P*_FOXP3+Tregs_ = 0.043, *P*_MPI_ = 0.028), while there were no significant differences in sTILs count, PD-L1 expression and MVD between primary tumors and metastatic lymph nodes.

### Univariate and Multivariate Analyses of pCR

From the binary logistic univariate analysis of patient age, T stage, N stage, histological grade, Ki67, preNAC sTILs (Pre-sTILs), preNAC CD8+ T cells (Pre-CD8+ T cells), preNAC CD4+ T cells (Pre-CD4+ T cells), preNAC FOXP3+ Tregs (Pre-FOXP3+ Tregs), preNAC PD-L1 (Pre-PD-L1), preNAC MVD (Pre-MVD), and preNAC MPI (Pre-MPI), the Pre-FOXP3+ Tregs count and Pre-MPI were found to be correlated with pCR (*P* < 0.05) (Table 4). From the multivariate analysis of histological grades, preNAC Ki67, Pre-sTILs, Pre-CD8+ T cells, Pre-CD4+ T cells, Pre-FOXP3+ Tregs, Pre-PD-L1, Pre-MVD and Pre-MPI, the Pre-FOXP3+ Tregs count, Pre-MPI and Ki67 were found to be correlated with pCR (*P* < 0.05) (Table 4).

**Table 4:** Univariate and multivariate analysis of pCR after NAC.

	**Univariate analysis**			**Multivariate analysis**		
**Parameter**	**OR**	**95%CI**	***P***	**OR**	**95%CI**	***P***
age	1.028	0.968–1.092	0.362	-	-	-
T stage	0.625	0.289–1.350	0.232	-	-	-
N stage	0.589	0.299–1.160	0.126	-	-	-
Tissue stage	1.968	0.439–2.134	0.936	0.499	0.176–1.417	0.192
Ki67	6.925	0.757–63.313	0.087	28.600	1.262–648.302	0.035
Pre-sTILs	0.739	0.042–12.872	0.836	2.220	0.014–357.134	0.758
Pre-CD8+T	0.994	0.946–1.045	0.825	0.879	0.767–1.007	0.062
Pre-CD4+T	1.037	0.971–1.107	0.278	1.081	0.969–1.205	0.162
Pre-FOXP3+	1.109	1.019–1.206	0.016	1.210	1.057–1.386	0.006
Pre-PD-L1	0.618	0.901–1.261	0.456	0.461	0.087–2.447	0.363
Pre-MVD	1.066	0.901–1.261	0.456	0.927	0.730–1.177	0.533
Pre-MPI	273.009	3.583–20802.657	0.011	6275.715	18.587–2118883.492	0.003

We also analyzed the relationship between the baseline ratio of populations (Tregs/T cells) and pCR (Table 5), and the results showed that both Foxp3+/CD8+ and Foxp3+/CD4+ had a higher ratio in pCR group than in non-pCR group.

**Table 5:** Relationship between the baseline ratios of populations and pCR.

**Parameters**	**pCR group**	**Non-pCR group**	***P*-value**
Foxp3+/CD8+	1.15 ± 0.78	0.34 ± 0.52	0.001
Foxp3+/CD4+	1.64 ± 1.58	0.44 ± 0.61	0.001

### Survival Analysis

**Figure 3:**
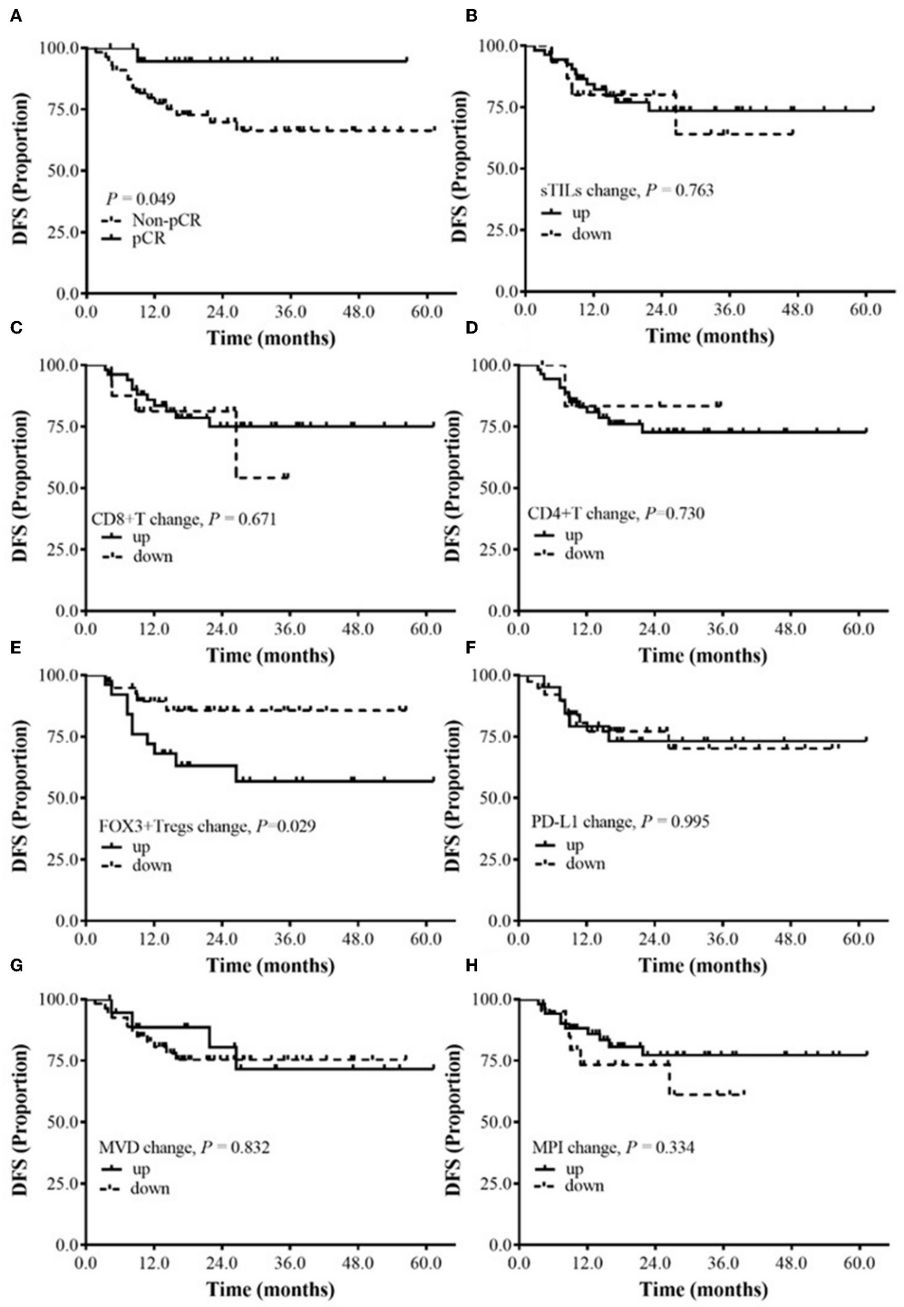
The relationship between the changes of sTILs, PD-L1, MVD, MPI, and DFS before and after NAC. **(A)** Survival analysis between pCR group and Non-pCR group. **(B)** Survival analysis of sTILs change. **(C)** Survival analysis of CD8+T change. **(D)** Survival analysis of CD4+T change. **(E)** Survival analysis of FOXP3+ Tregs change. **(F)** Survival analysis of PD-L1 change. **(G)** Survival analysis of MVD change. **(H)** Survival analysis of MPI change.

By the time of data analysis (June 1, 2019), the median follow-up time for the 75 patients with BC was 23.2 mo (6.1–64.5 mo), the median follow-up time for the patients in the pCR group was 22.3 mo (9.9–61.4 mo), and the median follow-up time of the patients in the Non-pCR group was 24.9 mo (6.1–64.5 mo). During the follow-up period, 17 patients (22.7%) had disease progression (7 cases of Her2-positive BC, 5 cases of luminal/HER2-positive BC, 2 cases of luminal/HER2-positive BC, 3 cases of TNBC); among them, 1 case was from the pCR group, and 16 cases were from the Non-pCR group. The data for survival analysis were immature and could not be used to calculate the median DFS of the patients. According to the preliminary data analysis, the DFS in the pCR group was significantly longer than that in the Non-pCR group (*P* = 0.049) (Figure 3A).

Subgroup analysis showed that after NAC, the DFS of BC patients with reduced FOXP3+ Tregs was significantly better than that of BC patients with increased FOXP3+ Tregs (*P* = 0.029) (Figure 3E). Currently, no correlation was found between DFS and the changes in sTILs, CD8+ T cells, CD4+ T cells, PD-L1 expression, MVD and MPI after NAC (Figures 3B–D,F–H).

**Figure 4:**
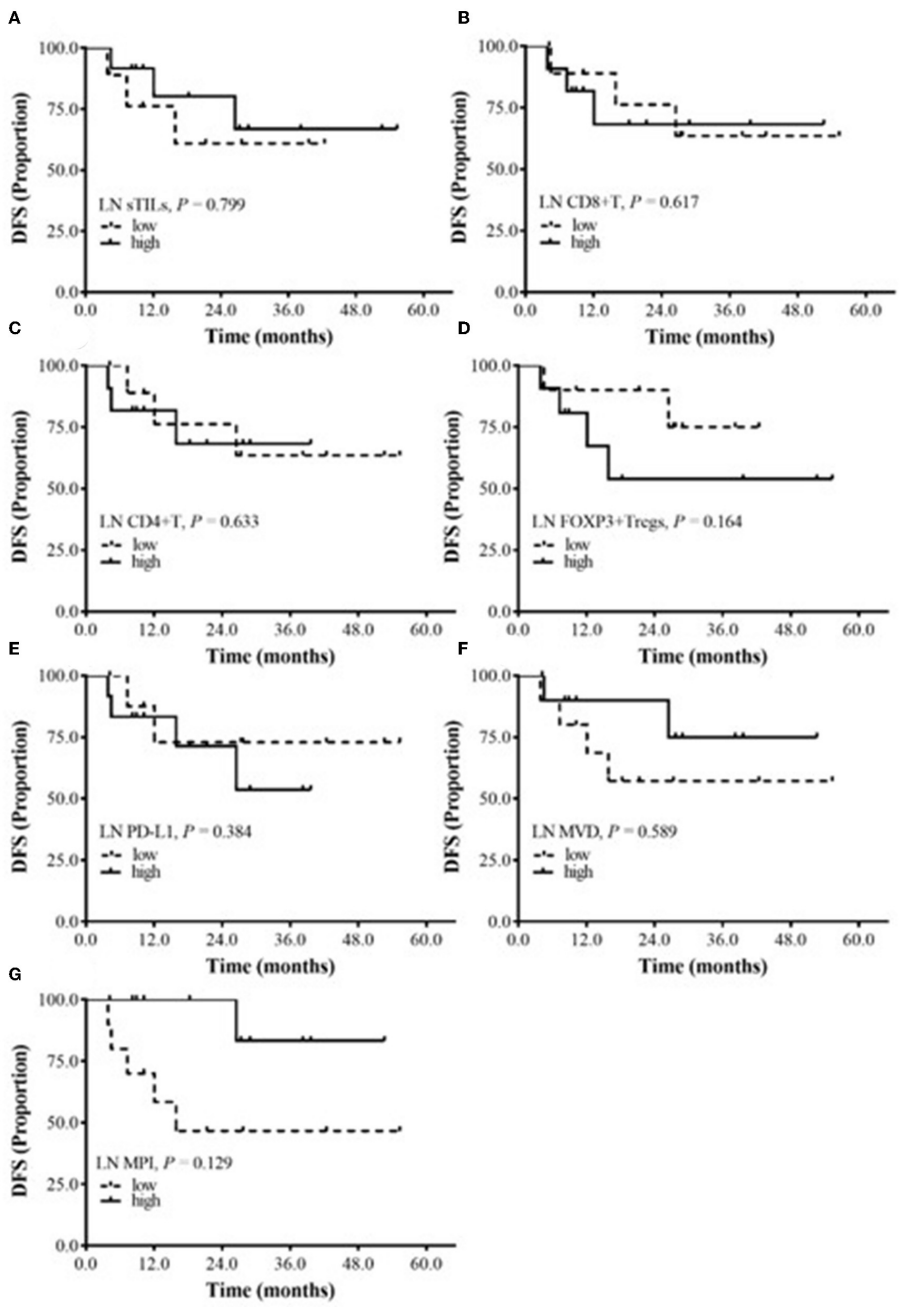
The relationship between DFS and sTILs, PD-L1, MVD, and MPI in Metastatic lymph node (LN). **(A)** Survival analysis of LN sTILs. **(B)** Survival analysis of LN CD8+ T cells. **(C)** Survival analysis of LN CD4+ T cells. **(D)** Survival analysis of LN FOXP3+ Tregs. **(E)** Survival analysis of LN PD-L1. **(F)** Survival analysis of LN MVD. **(G)** Survival analysis of LN MPI.

The relationship between sTILs and their subtypes, PD-L1 expression, MVD and MPI in metastatic lymph nodes and the prognosis of BC patients after NAC was explored. The median values were used to divide patients into high-expression and low-expression groups. No significantly correlation was found between DFS and the total sTILs, FOXP3+ Tregs, CD8+ T cells and CD4+ T cells; PD-L1 expression; or MPI and MVD in BC patients (*P* > 0.05) (Figure 4).

## Discussion

This study showed that after BC patients underwent NAC, sTILs, CD8+ T cells, CD4+ T cells and MPI increased, and FOXP3+ Tregs, PD-L1 expression and MVD decreased. The increase in sTILs and the decrease in PD-L1 expression were consistent with the results of Pelekanou et al. (22). However, Pelekanou et al. did not classify TILs, while we analyzed the subtypes of BC and found that the reduction in FOXP3+ Tregs was most prominent in patients with Her2-positive BC. NAC changed the number of sTILs in the tumor bed. Whether this change is correlated with pCR and DFS of patients has rarely been studied. Our study found that the percentage of patients with increased sTILs, CD8+ T cells, and CD4+ T cells was significantly higher in the pCR group than the Non-pCR group, while the percentage of patients with decreased FOXP3+ Tregs was significantly higher in the pCR group than the Non-pCR group. Further analysis of DFS showed that a decrease in FOXP3+ Tregs was significantly correlated with DFS. These results suggest that the increase in sTILs, CD8+ T cells and CD4+ T cells and the reduction in FOXP3+ Tregs might be related to the efficacy of NAC in BC patients. If the changes in these factors in tumor tissue can be dynamically detected during NAC, they may become predictive factors for the efficacy of NAC for BC. We did not find the significant difference of TILs between HER-2 positive and HER-2 negative BC, although all HER-2 positive BC patients received Trastuzumab treatment that was related to TILs (23). More study should be developed to focus this topic.

In addition to TILs, the vascular normalization-related indicators MPI and MVD were also examined in the present study. The results showed that after NAC, MPI was increased in all BC subtypes, suggesting that NAC can promote tumor vessel normalization. Meanwhile, the reduction in MVD was consistent with the reduction in FOXP3+ Tregs and PD-L1 expression, and the three may have synergistic effects. Tumor vessels often have abnormal structural and functional manifestations, such as a low MPI, an uneven basement membrane, and loose cell-to-cell connections. These abnormalities impair blood perfusion and drug delivery and increase the level of hypoxia in tumors and limit extravasation of T cells, especially cytotoxic T lymphocytes (CTLs) (24). Tumor vessel normalization enhances T cell infiltration and function. Tian et al. reported that effector CD4+ T cells supported vascular normalization and emphasized the interaction between blood vessels and T cells in cancer (25). Tolaney et al. studied the response of vascular density and normalization in BC patients to neoadjuvant bevacizumab and chemotherapy and found that a high baseline MVD in BC patients might be a necessary condition for bevacizumab-induced vascular normalization. Moreover, they found that an increased density of pericyte-covered microvessels was correlated with enhanced vascular function and oxygenation (20). The present study showed that MVD decreased and MPI increased after NAC, further confirming that the number of pericytes was inconsistent with the MVD. These results indirectly indicate that NAC promotes vascular normalization to some extent, enhances infiltration of CD8+ T cells and CD4+ T cells, and reduces infiltration of FOXP3+ Tregs.

Analysis of the correlation between sTILs in primary tumors and metastatic lymph nodes after NAC showed that CD8+ T cells, CD4+ T cells, FOXP3+ Tregs and MPI in primary tumors were higher than those in metastatic lymph nodes. From further analysis of the effects of them in the positive lymph nodes on the prognosis of BC patients, we found that the DFS of BC patients with low FOXP3+ Tregs and high MPI in the metastatic lymph nodes was better than that of patients with high FOXP3+ Tregs and low MPI; however, the difference was not statistically significant. This may be due to the small sample size in this study. Currently, no study has investigated sTILs and PD-L1 expression in primary tumors and metastatic lymph nodes after NAC in BC patients. The present study found that after NAC, the levels of CD8+ T cells, CD4+ T cells, FOXP3+ Tregs, and MPI in primary tumors were higher than those in metastatic lymph nodes, suggesting that the sensitivity of primary tumors to NAC may be different from the sensitivity of metastatic lymph nodes to NAC. These results need further validation. In addition, currently, few studies have assessed TILs in metastatic lymph nodes, and a standard assessment method is lacking. Kaewkangsadan et al. were the first to evaluate the abundance of TILs, Tregs (FOXP3+, CTLA4+) and NK cells in metastatic axillary lymph nodes (26). The evaluation method for TILs in metastatic lymph nodes still requires further exploration, and a unified standard is lacking for determining the TILs threshold, which requires the consensus of many researchers. In addition, evaluation of TILs in primary tumors and metastatic lymph nodes after NAC may provide a basis for more accurate prediction of patient prognosis and thus aid in selection of an optimal adjuvant treatment regimen.

This study is a relatively comprehensive study that explored the effect of NAC administered to BC patients on sTILs infiltration and PD-L1 expression in the TME of BC. However, this study still has drawbacks. First, the overall sample size of this study was small, leading to a low number of cases for each BC subtype, which might bias the results. Second, this study had a short follow-up period, with a median follow-up time of fewer than 5 years, and thus, the data for survival analysis were not yet mature. Third, the present study used core needle biopsy specimens before NAC to evaluate sTILs, PD-L1, MVD, and MPI, which might not be able to accurately reflect the those parameters in the entire tumor. In a previous study, Cha et al. (27) evaluated the consistency between TILs scores in tissue specimens from a core needle biopsy and TILs scores of resected BC specimens and found that the TILs scores of the core needle biopsy specimens were reliable in reflecting the TILs status of the entire surgically resected tumor.

## Conclusion

The baseline sTILs, FOXP3+ Tregs, and MPI in breast tumors can be used as predictive factors for the efficacy of NAC for BC patients. NAC increases infiltration of cells with immunological effects and reduces infiltration of immunosuppressive FOXP3+ Tregs and PD-L1. Changes in FOXP3+ Tregs infiltration and MPI may be used as prognostic markers in BC patients.

## Data Availability Statement

The raw data supporting the conclusions of this manuscript will be made available by the authors, without undue reservation, to any qualified researcher.

## Ethics Statement

The studies involving human participants were reviewed and approved by Huazhong University of Science and Technology Institutional Ethics Committee. The patients/participants provided their written informed consent to participate in this study.

## Author Contributions

QX analyzed the data and carried out the experiment. LY, TH, YC, and JW did data collection and structure determination. QW, XN, and JC designed the study and revised the manuscript.

### Conflict of Interest

The authors declare that the research was conducted in the absence of any commercial or financial relationships that could be construed as a potential conflict of interest.
