# Corrigendum: Changes in Tumor-Infiltrating Lymphocytes and Vascular Normalization in Breast Cancer Patients After Neoadjuvant Chemotherapy and Their Correlations With DFS

**DOI:** 10.3389/fonc.2021.661782

**Published:** 2021-03-01

**Authors:** Qiong Wang, Qun Xiang, Lan Yu, Ting Hu, Yangyang Chen, Jue Wang, Xiu Nie, Jing Cheng

**Affiliations:** ^1^ Cancer Center, Union Hospital, Tongji Medical College, Huazhong University of Science and Technology, Wuhan, China; ^2^ Department of Pathology, Union Hospital, Tongji Medical College, Huazhong University of Science and Technology, Wuhan, China

**Keywords:** breast cancer, tumor-infiltrating lymphocyte, neoadjuvant chemotherapy, microvessel pericyte coverage index, FOXP3+ Tregs

In the published article, there was an error in affiliations. Instead of “1 Cancer Center, Tongji Medical College, Union Hospital, Huazhong University of Science and Technology, Wuhan, China, 2 Department of Pathology, Tongji Medical College, Huazhong University of Science and Technology, Union Hospital, Wuhan, China”, it should be “1 Cancer Center, Union Hospital, Tongji Medical College, Huazhong University of Science and Technology, Wuhan, China, 2 Department of Pathology, Union Hospital, Tongji Medical College, Huazhong University of Science and Technology, Wuhan, China”

The authors apologize for this error and state that this does not change the scientific conclusions of the article in any way. The original article has been updated.

